# Aphrodisiac Use Associated with HIV Infection in Elderly Male Clients of Low-Cost Commercial Sex Venues in Guangxi, China: A Matched Case-Control Study

**DOI:** 10.1371/journal.pone.0109452

**Published:** 2014-10-06

**Authors:** Zhenzhu Tang, Xinghua Wu, Guojian Li, Zhiyong Shen, Hongman Zhang, Guanghua Lan, Xue Feng, Rui Lin, Abu S. Abdullah, Zunyou Wu, Cynthia X. Shi

**Affiliations:** 1 Guangxi Zhuang Autonomous Region Center for Disease Control and Prevention, Nanning, Guangxi, China; 2 Department of HIV/AIDS Control and Prevention, Guangxi Zhuang Autonomous Region Center for Disease Control and Prevention, Nanning, Guangxi, China; 3 Department of Pharmaceutical Systems and Policy, West Virginia University, Morgantown, West Virginia, United States of America; 4 Department of Medicine, Boston University School of Medicine, Boston, Massachusetts, United States of America, and School of Public Health, Guangxi Medical University, Nanning, China; 5 National Center for AIDS/STD Control and Prevention, Chinese Center for Disease Control and Prevention, Beijing, China; 6 Chinese Center for Disease Control and Prevention, Beijing, China, and School of Public Health, Harvard University, Boston, Massachusetts, United States of America; UNC Project-China, China

## Abstract

**Background:**

Rising HIV infection rates have been observed among elderly people in Guangxi, China. Inexpensive aphrodisiacs are available for purchase in suburban and rural areas. This study aims to investigate the association between aphrodisiac use and increased HIV risk for middle-aged and elderly men in Guangxi.

**Methods:**

A matched case-control study of aphrodisiac use-associated HIV infection was performed among male subjects over 50 years old who were clients of low-cost commercial sex venues in Guangxi. The cases were defined as clients who were HIV-positive and two controls were selected for each case. The cases and the controls were matched on the visited sex venue, age (±3 years), number of years of purchasing sex (±3 years), and educational attainment. Subjects were interviewed and tested for HIV. Paired t-test or McNemar Chi-squared test were used to compare the characteristics between the cases and controls. A stepwise conditional logistic regression was used to identify risk factors associated with HIV infection.

**Findings:**

This study enrolled 103 cases and 206 controls. Aphrodisiac use (P = 0.02, odds ratio (OR) = 1.81, 95% CI = 1.08–3.04), never using condom during commercial sex encounter (P = 0.03, odds ratio (OR) = 1.82, 95% CI = 1.08–3.07), and lacking a stable partner (P = 0.03, odds ratio (OR) = 1.76, 95% CI = 1.05–2.98) were found to be risk factors for HIV infection among the study groups. For subjects reporting aphrodisiac use, the frequency of purchasing sex was positively correlated with the frequency of aphrodisiac use (r = 0.3; p = 0.02).

**Conclusions:**

Aphrodisiac use was significantly associated with increased HIV infection risk in men over 50 years old who purchased commercial sex in the suburban and rural areas of Guangxi. Further research and interventions should address the links between aphrodisiac use, commercial sex work, condom use, and increased HIV transmission.

## Background

The HIV/AIDS epidemic is posing a greater public health threat in China with a total of 434,000 detected cases of HIV/AIDS as of September 2013. Heterosexual transmission accounted for 70% of the newly reported cases in China in 2013 [1]. In recent years, the HIV epidemic in China has expanded from high-risk groups to the general population [2]. Guangxi, a province in southern China, ranks second among China’s 31 provinces in terms of cumulative reported HIV/AIDS cases [3]. Guangxi province shares a border with Vietnam and the “Golden Triangle”, which is an entry point for drug trafficking routes and cross-border migration of sex workers. This has led to a relatively high number of commercial sex behaviors in cities within Guangxi [4]. Prior to 2000, HIV transmission occurred mainly through intravenous drug use. However, over the last 15 years, heterosexual transmission has gradually become the primary route of infection in Guangxi, accounting for 3.9% of new cases in 1997 [5], 37.1% in 2006 and 90.0% in 2011 [6].

In recent years, an increasing HIV infection rate has been observed among elderly people in Guangxi. Individual over 60 years old accounted for 18.7% of the total HIV cases in 2009 and this number increased to 28.4% in 2011 [6], which includes cases of individuals over 85 years old. Additionally, the majority of the cases involving elderly patients were from rural areas, which raised concerns for public health officials [5], [6]. Previous studies reported unsafe commercial sex as an important risk factor of HIV infection in this age group [7]–[10]. A 2011 study in Nanning, the capital city of Guangxi, showed that heterosexual transmission accounted for 90% of the HIV cases among those aged over 50 years old. This study also reported low-cost commercial sex venues as the primary sites of infection among older adults. Given the wide availability of low-cost commercial sex venues in Guangxi, purchasing sex from these venues by older adults is common [11]–[14]. In this study, a low-cost commercial sex venue is defined as a site where the price for a single sexual intercourse is less than US $6. Examples of such sites include inexpensive hotels, places with rooms rent by the hour, or outdoor settings.

Erectile dysfunction is more common among elderly men than young men; thus, erectile dysfunction medications (EDMs) have become popular among older men to enhance sexual desire and performance [15]. EDMs can enhance erections, intensify sexual desire, and prolong sexual intercourse [16], [17]. Sildenafil, an oral medication sold under the trade name Viagra, is one of the most commonly used EDMs. Studies have reported high rates of EMD use among men who have sex with men [18], [19] and elderly men [20]. In the United States, among men older than 50, rising rates of gonorrhea and HIV transmission through heterosexual contact are paralleled by increases in the number of sildenafil prescriptions [21]. There are reported associations between EDMs, increased risk of STDs [18], and more frequent use of recreational drugs [19]. Viagra was introduced into China in July 2000 and has been available in Chinese retail drugstores from 2004. The price of Viagra is about US $15 per pill, which is prohibitively expensive for most rural residents in Guangxi. Inexpensive alternative aphrodisiacs, which are purported to have similar effects as sildenafil, are widely available in suburban and rural areas in Guangxi. These low-cost drugs are manufactured in unregulated illegal workshops and are often sold with no information about their chemical composition. Little is known about aphrodisiacs use among in elderly people in China, particularly in rural areas. It is also uncertain whether HIV transmission is associated with aphrodisiac use in this population. Through a matched case-control design, our study aims to investigate the association between aphrodisiac use and HIV risk among men older than 50 years in Guangxi.

## Methods

### Ethics Statement

Ethical approval was obtained from Guangxi Institutional Review Board Composition. Written informed consent was obtained from all participants.

### Study design and participants

A matched case-control study was conducted in Guangxi, China. Cases and controls were selected from a cross-sectional sample of male clients of low-cost commercial sex venues. A total of 3485 men over 50 years were interviewed in Guangxi from October to December 2012. They came from thirteen different regions (Liuzhou, Guilin, Guigang, Beihai, Binyang, Luzhai, Duan, Daxin, Lingshan, Pingnan, Quanzhou, Lingchuan and Qintang) and these study sites were selected according to the apparent cumulative number of reported HIV-positive cases and the consideration of a geographical diversity of samples. The inclusion criteria of this cross-sectional study were that the male clients belonged to commercial sex venues in these thirteen regions and they were over 50 years old at the time we conducted this study. The survey response rate was 73.9% (3485/4716). Field surveys were primarily carried out in suburban and rural areas to reflect the geographical distribution of commercial sex venues. The cases were defined as male clients over 50 years old in low-cost commercial sex venues and were identified as HIV-positive through a Western blot or a nucleic acid test. Two controls were matched to each case on the following aspects: having visited the same or neighboring commercial sex venue, age (±3 years), number of years of purchasing sex (±3 years) and educational attainment. Given that very few subjects in case group were reported to use condom during every commercial sex encounter, and using condom during every commercial sex encounter was considered as a protective factor, cases and controls that reported condom use during every commercial sex encounter were excluded from the analysis.

The sample size of matched case-control study was calculated using the following formula: (P1 = P0RR/[1+P0(RR−1)], p = 0.5(P1+P0), q = 1−P0). P1 stands for the exposure rate in case group, P0 is the exposure rate in control group, RR is the relative risk of HIV infection associated with the exposure, p is the overall exposure rate, and q is the proportion of the non-exposure in control group. Given an estimated minimum exposure rate of 20% for elderly male clients who used aphrodisiacs, and a = 0.95, β = 0.10, expected OR = 2.5–3.5, matching ratio of case to control = 1∶2, the sample size then was calculated as 36–71 cases and 72–142 controls.

Blood samples were taken at interviews, and HIV infection tests were performed in sequential batches in the order of blood draws following the conclusion of the interviews. Consequently, neither participants nor study personnel were aware of the participants’ HIV infection status during the interview. To avoid selection bias, eligible controls were drawn from the same or neighboring venue in accordance with chronological interview order. Also, counseling on STDs and HIV prevention was performed after each interview.

### Variables and Measurements

Each eligible subject was interviewed to gather information on age, ethnicity, education, awareness and knowledge on HIV/AIDS, residency, relationship status, erectile dysfunction, aphrodisiac use, and the number of years of purchasing commercial sex. Aphrodisiac use was defined by 1) having used aphrodisiac; and 2) meeting at least one of the following criteria: having used aphrodisiacs at least once in the past month, taking aphrodisiacs 1–5 times or more in one month, being unable to achieve an erection without an aphrodisiac, or being unable to have penetrative intercourse without an aphrodisiac. The study participants reported of having a spouse, cohabiting partner, or regular sexual partner during the previous six months before the interview were defined as “having a stable partner”. A national Center for Disease Control and Prevention (CDC) standard questionnaire on HIV/AIDS knowledge were administered to the study participants and those who answered six of eight questions correctly were classified as “having awareness about HIV/AIDS”.

Blood serum samples were tested in local CDCs by using a commercially available screen test kit (Wondfo diagnostic kit for HIV1/2 antibody (Colloid al gold)) and an ELISA kit (Wantai HIV (1+2) Ab ELISA) at the same time. Western blot tests were conducted to confirm the positive HIV infection (MP Diagnostics; National Guideline for Detection of HIV/AIDS, China). Blood samples producing negative screening results were further tested at the Guangxi CDC by the HIV-1 RNA viral load test (COBAS AmpliPrep/COBAS aqMan HIV-1 Test, version 2.0; viral load over 5000 cp/ml). All serum samples were stored at −20°C.

### Data management and analysis

Data were double-entered in EPIDATA v3.1 with weekly supervision. A CHK quality control procedure was applied according to logical relationships. Descriptive statistics (mean and standard deviation) were calculated for continuous variables, e.g. number of years of purchasing sex. Age was categorized by decades. Frequencies and percentages were calculated for discrete variables, including age group, ethnicity, educational attainment, tobacco and alcohol use, condom use, aphrodisiac use, erectile dysfunction, having a stable partner, and frequency of purchasing commercial sex during the last month. The paired t-test or McNemar Chi-squared test was used to compare groups. A step-wise conditional logistic regression was used to identify risk factors associated with HIV infection. A risk factor was selected if the P value was less than 0.05. All analyses were performed using SPSS for Windows (version 18.0; IBM, NY, USA).

## Results

### Matched case-control study

The cross-sectional survey among 3485 subjects in conjunction with HIV test identified a total of 103 HIV-positive cases, of which 26 subjects were derived from suburban areas and 77 subjects from rural areas. As each case was matched with 2 controls, a total of 309 individuals were included in this study. Their ages ranged from 50 to 84 years with a mean of 63.6 years (SD, 8.4 years). The median years of purchasing sex of the study participants were 6 (IRQ, 4–10 years). 74.8% (231/309) had an educational attainment level lower than middle school. None of the participants reported condom use in every commercial sex encounter. Based on the criteria used to define aphrodisiac use, 43.7% (45/103) cases had used aphrodisiacs compared to 21.8% (45/206) of the control group, demonstrating that the cases had significant higher rate of aphrodisiac use than the controls (Table 1). Our study also found that 69 (67.0%) of case group and 115 (55.8%) of control group never used condom during commercial sex encounter. In addition, 57.3% (59/103) of cases and 68.9% (142/206) of control group participants had stable partners. Finally, 12.6% (13/103) of cases and 15.5% (32/206) of controls reported at least several symptoms of erectile dysfunction. There was no difference between the cases and controls in terms of ethnicity, suburban or rural residencies, erectile dysfunction, awareness about HIV/AIDS, tobacco and alcohol consumption, and frequency of purchasing commercial sex during last month (Table 1).

**Table 1:** Demographic Characteristics and Other Risk Factors for Cases and Controls.

Variables	Cases (% or SD)	Controls (% or SD)	P-value
Age ≥65	51(49.5%)	93(45.1%)	0.1
Han Ethnicity	63(61.2%)	124(60.2%)	1.0
Educational Attainment			1.0
Lower than middle school	74(71.9%)	157(79.2%)	
Middle school and above	29(28.1%)	49(23.8%)	
Residency			1.0
Rural	77(74.8%)	159(77.2%)	
Suburban	26(25.2%)	47(22.8%)	
Tobacco and Alcohol Use			0.7
Tobacco/alcohol/tobacco & alcohol	81(78.6%)	158(76.7%)	
None	22(21.4%)	48(23.3%)	
Condom use			0.01
Never use	69(67.0%)	115(55.8%)	
Use sometimes	34(33.0%)	91(44.2%)	
Years of purchasing sex	7.4±4.9	7.2±4.7	0.7
Aphrodisiac Use	45(43.7%)	45(21.8%)	0.03
Erection dysfunction	13(12.6%)	32(15.5%)	0.2
Having a stable partner	59(57.3%)	142(68.9%)	0.00
Frequency of purchasing commercial sexduring last month			0.7
< = 1	76(74.0%)	148(71.8%)	
>1	27(26.0%)	58(28.2%)	
Awareness of HIV/AIDS	61(59.2%)	136(66.0%)	0.4

Using a stepwise conditional logistic regression (Table 2), the risk factors associated with HIV infection were: aphrodisiac use (P = 0.02, odds ratio (OR) = 1.81, 95% CI = 1.08–3.04), never using condom during commercial sex encounter (P = 0.03, odds ratio (OR) = 1.82, 95% CI = 1.08–3.07), and not having a stable partner (P = 0.03, odds ratio (OR) = 1.76, 95% CI = 1.05–2.98).

**Table 2:** Risk Factors Associated with HIV Infection.

Risk Factor	Odds Ratio	P-value	95% Confidence Interval
Aphrodisiac use	1.81	0.02	1.08–3.04
Never using condom during each commercial sex encounter	1.82	0.03	1.08–3.07
No stable partner	1.76	0.03	1.05–2.98

### Characteristics of subjects who used aphrodisiacs

Among the 90 aphrodisiac users, 94.4% (85/90) reported that aphrodisiac use helped achieve erection (54.4%; 49/90), increased erection hardness (61.1%; 55/90), prolonged sexual intercourse (43.3%; 39/90), and increased sexual pleasure (20.0%; 18/90). About a quarter (26.7%; 24/90) of elderly subjects reported that they could not perform penetrative sex without using aphrodisiacs. 54.4% (49/90) reported they never used condom during every commercial sex encounter, and there was no difference between aphrodisiac users and none-aphrodisiac users for their condom use (data not shown). The frequency of purchasing commercial sex in the last month was positively correlated with the frequency of aphrodisiac use (r = 0.25; p = 0.02).

## Discussion

Our cross-sectional study among 3485 male subjects aged over 50 who purchased sex at the low-cost commercial sex venues in Guangxi, China enabled us to conduct a matched case-control study of 103 cases of HIV infection and 206 matched negative controls. This matched case-control study is the first to report that aphrodisiac use is significantly associated with an increased HIV risk for men over 50 years old who purchased commercial heterosexual sex. Also, never using condom during each commercial sex encounter and lacking a stable partner were found to be risk factors for HIV infection among this study group.

Aphrodisiacs, used as erectile dysfunction medications (EDMs) to help men achieve erection, increase erectile hardness, and prolong sexual intercourse [22], are widely available in China [23]. One-third of the participants in this study were considered users of aphrodisiacs by relatively strict criteria. Indeed, one study in Guangxi in 2013 showed that 30.2% of the male commercial sex clients over 60 years old used aphrodisiacs during commercial sex encounter [24]. Although use of aphrodisiacs is not widely recognized as a direct causal factor for HIV transmission, our present study has shown that the frequency of aphrodisiac use was correlated with the frequency of purchasing sex and, importantly, aphrodisiacs use was significantly associated with increased HIV risk in elderly men committing commercial heterosexual intercourse (Table 1). These findings are consistent with previous report that sildenafil, the active pharmaceutical ingredient of Viagra, was associated with increased heterosexual transmission of HIV and gonorrhea in men over 50 years old in the USA in that rising rates of heterosexually transmitted HIV and gonorrhea were in parallel to the escalating number of sildenafil prescriptions [21]. Moreover, the association between aphrodisiac use and increased HIV transmission in MSM has also been reported [19], [25].

How aphrodisiacs use significantly associates with increased HIV risk in elderly men who are clients in the low-cost commercial sex venues has not been fully understood. Presumably, aphrodisiac may cause aphrodisiac users to exchange more body fluids during intercourse than non-aphrodisiac users due to its effect on prolonging sexual intercourse. This may lead to a higher probability of HIV transmission, particularly during unprotected sexual intercourse. While a high percentage of the subjects reported that they could not perform penetrative sex without using aphrodisiacs, it is possible that, the aphrodisiacs use may not be a causal factor for HIV transmission, but rather a marker of a high-risk lifestyle for HIV transmission and infection in elderly male clients of commercial sex.

We also found that never using condom during each commercial sex encounter and lacking a stable partner were independently associated with increased HIV risk for middle-aged and elderly males. Men without stable partners, particularly elderly men, are more likely to purchase commercial sex and to use aphrodisiacs. Not using condom during each commercial sex encounter resulted in possible exposure to HIV virus, which increased HIV infection risk. These risk factors are linked and may have interactive effects on HIV transmission risk.

The present study has a few limitations. First, there may be recall bias and reporting bias similar to those of the other retrospective case-control studies. Second, there might be biases in the sampling of cases and controls due to the fact that individuals who were diagnosed of HIV infection in advance were more likely to decline study participation. Third, the temporal ambiguity bias that an individual used aphrodisiacs after having infected with HIV could not be excluded. Condom use during every commercial sex encounter was excluded from the analysis, which prohibited the investigation of association between condom and aphrodisiacs use. Further study was required to address this issue. Therefore, we intend to follow the HIV-negative subjects in half of the study sites through annual behavioral questionnaires and blood sample test in a hope of obtaining data helpful for addressing these questions.

A recent study in one of our study sites showed that most of the injecting drug users aged 15 to 49 years [26], and the average age of men who had sex with men was 28.1±8.2 years reported in a cohort study in the capital city of Guangxi [27], which supported that the route of HIV transmission among those over 50 years old in Guangxi was more likely to be heterosexual contact, given that the participants in this study were interviewed near the low-cost commercial sex venues. Unexpectedly, 68.0% of the subjects in the case group in this study interviewed at the low-cost commercial sex venues were over 60 years old. This strongly suggests that the upper limit of age for routine HIV testing in this region be evaluated to cover more elderly people under surveillance for HIV infection.

Our study of older men in suburban and rural areas in Guangxi has demonstrated that the use of aphrodisiacs was associated with increased HIV risk. We suggest that further research and public health interventions be required to address the links between aphrodisiac use, commercial sex work, condom use, and increased HIV transmission.
